# Re-imagining transdisciplinary education work through liminality: creative third space in liminal times

**DOI:** 10.1007/s13384-022-00519-2

**Published:** 2022-04-07

**Authors:** Giedre Kligyte, Adrian Buck, Bem Le Hunte, Sabrina Ulis, Amanda McGregor, Beth Wilson

**Affiliations:** TD School (Transdisciplinary School), University of Technology Sydney, Sydney, Australia

**Keywords:** Transdisciplinary learning, Work integrated learning, Liminality, Third space, Mutual learning

## Abstract

This study draws on the tradition of transdisciplinarity to extend the boundaries of interdisciplinary educational work. In this paper, we apply the concepts of liminality and third space to examine a case of a professional immersive experience (PIEx), designed in response to the catastrophic disruption of work-integrated learning opportunities by the COVID-19 pandemic. The study uses a participatory reflexive methodology to interrogate the range of ways liminality was manifest in PIEx. First, we examine liminal learning in the virtual environment, which facilitated the unfolding of connections between different spaces, locations and people. Second, we seek to understand the PIEx experience through the concept of third space, highlighting the fluidity of roles, where educators, students and industry partners generate new knowledge and practices together. Lastly, we examine the experience through the boundary-crossing lens of transdisciplinarity. We conclude by gesturing towards a new understanding of work integrated learning, as it could take place in the future, well beyond the walls of the university.

## Introduction

Over recent decades, higher education institutions around the world have undergone significant transformations. The historical university concern with education and scholarship has been expanded to include considerations for social impact. In Australia and around the globe, universities are increasingly required to demonstrate the employability of their graduates, create socially relevant knowledge and provide tangible benefits to external communities (Barnett, 2017; Collini, 2012; Davis, 2017). In this context, the nature of academic labour is transforming from being primarily governed by academic norms to serving a multiplicity of social purposes and goals (Barnett, 2017; Leisyte & Dee, 2012).

Transdisciplinarity is one approach that some universities are experimenting with to create education outcomes and knowledge that are relevant to complex contemporary challenges. Transdisciplinary scholars and practitioners seek to create positive impact in the world by transcending disciplinary boundaries and practices, including integrating academic knowledge with other knowledge types, such as local, practical and Indigenous knowledges (Jantsch, 1972; Klein, 2004; Polk & Knutsson, 2008). Transdisciplinary approaches start “without a particular disciplinary strategy in mind” (Manderson, 1998, p. 66); they co-evolve based on the challenge at hand, shaped by what is on and who is at the table (Baumber et al., 2020; Kligyte et al., 2021; van der Bijl-Brouwer et al., 2021). Transdisciplinarity initially gained prominence in sustainability-related fields (Mitchell et al., 2015; Polk & Knutsson, 2008), but it is now being applied in numerous other contexts such as urban environments (Ramadier, 2004), design (Crosby et al., 2018) and education (Baumber et al., 2020; Kligyte et al., 2019).

In this paper, we, a heterogeneous group of educators and practitioners situated in a pan-university TD School (Transdisciplinary School) at University of Technology Sydney, discuss our work in enabling transdisciplinary learning across porous university boundaries. This education work is a complex and distributed endeavour, which requires us to orchestrate expertise from across a range of scholarly areas and practical domains. It takes place in between disciplines, roles and organisations, and often leads to significant, even transformational learning outcomes for those involved (Kligyte et al., 2019). In this paper, we lean on the anthropological concepts of *liminality* and *third space* to examine these types of encounters.

We begin by highlighting the links between transdisciplinary education work and the Work Integrated Learning (WIL) literature, outlining the disruption of WIL opportunities by the COVID-19 pandemic. We introduce the concepts of liminality and third space, followed by an explanation of the reflexive methodology used in this study. By examining the case of a professional immersive experience (PIEx), we interrogate the range of ways liminality was manifest in this experience. We discuss new insights and avenues for action opened up by the idea of liminality in education work and conclude by highlighting the implications to education scholars and practitioners.

## Designing immersive professional experiences during the pandemic

Nowhere is the labour of engagement with diverse groups of people more apparent than in transdisciplinary WIL settings. Interest in WIL has grown among higher education researchers over recent years, partly, due to the increasing pressure for universities to graduate ‘job-ready’ professionals. As an educational approach, WIL seeks to enable students to “integrate theory with the meaningful practice of work” through “relevant work-based experiences” (International Journal of Work-Integrated Learning, 2021). WIL involves at least three types of stakeholders: students, universities and partner organisations, in creating learning opportunities that feature “authentic and meaningful work-related tasks” (International Journal of Work-Integrated Learning, 2021). WIL is typically offered and accredited by university providers, but student learning is primarily derived from engagement with supervisors in professional or practical contexts.

McRae et al. (2018) highlight that since a variety of stakeholders contribute to WIL experiences, individuals can have divergent expectations about meaningful WIL. These differing perspectives are shaped by individuals’ past experiences and social perceptions about the value of WIL. Thus, to create coherent WIL experiences, a range of goals and motivations have to be negotiated by academics, professionals and individuals situated in partner organisations. Whilst providing high-quality WIL is challenging at the best of times, in 2020, WIL opportunities were particularly disrupted by the COVID-19 pandemic. The traditional WIL models reliant on physical work placements, such as internships, clinical placements or practicums had to be reimagined for online delivery almost overnight (see these journal special issues focussed on university responses to the pandemic, Kay et al. (2020), Green et al. (2020), and Devlin and McKay (2021)).

The experience of the COVID-19 pandemic touched all sectors of society in different ways. University staff members were compelled to draw on their collective resilience and creativity to continue providing high-quality learning experiences amidst the disruption (Baumber et al., 2021). The effects of the pandemic were simultaneously felt by the WIL partner or host organisations. Just like universities, businesses were grappling with uncertainty and the need to adapt to the ever-evolving effects of the pandemic. At the same time, students were experiencing tremendous challenges, such as the loss of employment and income whilst dealing with the social and mental health impacts of lockdowns. The pandemic truly exemplified how “contemporary problems in an increasingly globalised world do not fit neatly within traditional disciplinary or institutional boundaries” (Fitzgerald et al., 2020), and how new ways of thinking about education work were needed.

The question about what constitutes meaningful WIL engagement for the various stakeholders was brought to the fore by the rapid pivot to online teaching. Whilst the transdisciplinary WIL offering by TD School, has always entailed aspects of co-creation, the unprecedented context of the pandemic created a new impetus for the definition of meaningful WIL experience to be co-explored, co-created and co-defined. The transdisciplinary approach taken to conceive PIEx enabled us to create a reflexive liminal space for this exploration of meaning. The concepts of liminality and third space outlined in the next section were central to this reflexive process, helping us to illuminate our transdisciplinary education work in new ways, and rise above knee-jerk emergency pedagogy.

## Liminality in transdisciplinary education

Liminality is a kind of flux. […] it renders things fluid, less certain than they used to be, and starts to transform the learner (Land et al., 2014, p. 1).

There is an extensive literature exploring the concept of liminality in learning (Land et al., 2014). Drawing on the discipline of anthropology, where liminality is used to describe the transitory states in initiations (Turner, 1967), liminality is now often used in education to highlight the sense of instability and the prolonged process of oscillation between old and new understandings routinely experienced by learners. Liminality in learning is frequently discussed in negative terms, as “uncertainty, confusion, and lack of confidence” (Keefer, 2015, p. 18) or as “a suspended state in which students sometimes can struggle to cope” (Land et al., 2014, p. 1). To progress through a liminal state, learners have to let go of certain aspects of past learning and identities which can feel unsettling and confronting (Land et al., 2014). Much of the research into liminal states in learning seeks to build strategies for safely and effectively guiding students through this uncertainty so that they can emerge on the other side transformed and matured (for example, see Meyer & Land, 2005).

Conversely, liminal states can also be seen as creative spaces—“realm[s] of pure possibility whence novel configurations of ideas and relations may arise” (Turner, 1967, p. 97). Liminality necessarily implies fluidity, offering “a provisional, exploratory space with plenty of unexplored possibilities, where things [can be] held in tension—an almost perpetual liminal state of creativity” (Land et al., 2014, p. 2). The creative possibilities attendant to liminal states relate to the notion of third space advanced by critical theorist Homi Bhabha (1994). Third spaces are heterogeneous contexts where conventional hierarchies, boundaries and delineations are diminished:

Third space is an interstitial realm, like the threshold, which accommodates ambivalence, conflict, confusion, movement, change and notably, potentiality. It is held open by the tension between different spaces and temporalities and generates relationships in which both sides are changed through the negotiation of incommensurable strategies, rules and identities in cultural processes and practices. (Engels-Schwarzpaul & Emadi, 2011, p. 1)

Generative third spaces embrace diversity, difference, experimentation, participation and co-creation to stimulate new ways of thinking and creativity. They are “risky place[s] on the edge […] but also with new possibilities” (Soja & Hooper, 1993, p. 190). In third spaces, “things can be re-thought [and] re-authored […] one can undo the script of self and re-script” (Land et al., 2014, p. 5).

Conceptualising transdisciplinary WIL as a liminal space opens up new possibilities for articulating the work involved in creating WIL experiences that are meaningful to the various stakeholders and students. To establish the practical context for the exploration of liminality, we now introduce the PIEx case study.

### The case study: PIEx

**Table:** 

Usually, education happens within the boundary of the university or within the four walls of a classroom, and it's very contained. In a liminal space like PIEx, we engage with industry partners. We let students loose on industry briefs, we give them opportunities to work across and beyond the boundaries of the university. It’s about not seeing learning as an institutionalised thing; it's not siloed, it's not separated. We think of it as a porosity of university education in this third space where industry partners, students and university staff work together as equals to generate new knowledge and responses to the challenges in the world. (Bem, Course Director)	

The PIEx transdisciplinary WIL offering was designed as a COVID-19 pandemic response to replace a 3-week-long intensive innovation internship within the Bachelor of Creative Intelligence and Innovation (BCII) degree at University of Technology Sydney (UTS). BCII is a combined degree that brings together diverse knowledges from across 25 different undergraduate programs at UTS. Over the first 3 years, students undertake intensive subjects exploring transdisciplinary approaches alongside their ‘core degree’ (e.g., in business, science, communications or other fields). In the fourth and final year, students fully immerse themselves in transdisciplinary learning, completing an internship, two long-term ‘capstone’ projects and subjects supporting students in articulating their transdisciplinary capabilities to a range of audiences, such as future employers. The hallmark of the BCII curriculum from the very first year is opportunities for students to work on real-world challenges in partnership with industry, community or government stakeholders.

PIEx was developed as a transdisciplinary WIL offering seeking to provide an enriching, immersive and authentic learning experience during the pandemic. The learning objectives of the Professional Immersive Experience (PIEx) were comparable to those of an internship, specifically, focussing on enhancing students’ professionalism, problem solving and understanding of organisational innovation practices. PIEx was envisioned to be a bespoke, virtual-workplace simulation co-designed with external partners, academics, students and professionals. To cater for a diverse range of student disciplines and interests, a choice of professional streams was offered for students to participate in. Each stream involved a different industry partner as the ‘employer’, a unique brief on an emerging issue and access to a range of experts across industry for knowledge and guidance. In doing so, PIEx sought to immerse students in a role similar to one they may apply for after graduation, exploring key stages at the beginning of employment, including recruitment, induction, team building and delivering projects.

A broad call for Expressions of Interest was circulated by the industry partnerships team within the TD School industry partner network. The call received responses from 28 organisations, and additional strategic partners were approached with bespoke proposals. Two professional streams were established: a living, learning workplace challenge with a large corporate consultancy as the ‘employer’, and a challenge centred around setting up communities of practice in Sydney’s Peri-Urban areas led by an Australian Government funded agency. An additional four partners agreed to participate as ‘clients’, providing day-long briefs aligned with the context of exploration within a professional stream. Further, 48 individual partners contributed as mentors in professional streams or as workshop leaders and panellists exploring inclusive practices, recruitment, stakeholder engagement and teamwork. In total there were 89 individuals from the external partner network involved in PIEx.

### PIEx subject design

The curriculum of PIEx was designed around five phases deemed to be essential for students experiencing an internship, with appropriate external stakeholders engaged for each phase:

1. Recruitment.
2. Induction and workplanning.
3. Real-time project work.
4. Completion.
5. Debrief.

Key subject design innovations were centred around creating space in the curriculum for various stakeholders to come together at the boundaries of the well-established practices, roles and identities. First, in the opening workshop, students and industry partners were invited to share and discuss their reasons for participation in the subject. Drawing on the transdisciplinary learning cycle (Muller et al., 2005, cited in McGregor, 2017), all stakeholders were encouraged to challenge their preconceptions, reflect upon the personal truths of other participants, and consider whether a shift in their own mindsets was needed. Through this, PIEx created a space where all stakeholders were invited to articulate, negotiate and align their aims for this WIL experience. The subject finished with a debrief workshop, inviting the participants to reflect on their actual PIEx experiences and achievements.

Second, as in all transdisciplinary learning experiences, non-academic PIEx participants and peers made a substantial contribution to students’ learning, with a great deal of relational capital and trust circulating through the newly formed networks. The transdisciplinary education work called for fluid identities and capabilities that were ‘neither here or there’: they did not sit neatly in typical academic or professional role descriptions. A new, boundary-crossing stream leader role was created to guide the students through the subject, undertaken by two academic tutors with extensive industry experience. The stream leaders were entrusted to design and oversee the industry project component, as well as take the role of a manager and mentor for the duration of the industry challenge.

Finally, inclusive practice was central to the design of PIEx. The academic deliverables primarily centred around students’ understanding of and effectiveness in planning, creating, progressing and refining inclusive learning and work environments. Through a focus on inclusivity, the experiential project work and reflexive assessment tasks mutually informed each other, continually challenging students to reframe their own views based on encounters with different perspectives.

## The reflexive and dialogic approach

The authors of this paper have all been variously involved in the co-creation of PIEx: three of the authors are members of the Industry Partnership Team, with the remaining three authors being the PIEx Subject Co-ordinator, the BCII Course Director and a Senior Lecturer in Transdisciplinary Education at TD School. Industry partner, casual staff member and student perspectives were included through the PIEx debrief workshop, which constituted part of the research data outlined below.

In addition to co-creating an inspiring transdisciplinary WIL offering in unprecedented times, the PIEx team engaged in ongoing reflexive conversations. Building on a pragmatist research tradition, the intent was to challenge the perceived “dichotomies between *understanding* and *practice”* (Popa et al., 2015, p. 48) and integrate deliberate acts of sense-making into our work. Building on Polk’s (2015) definition of reflexivity as “on-going scrutiny of the choices that are made when identifying and integrating diverse values, priorities, worldviews, expertise and knowledge” (p. 114), reflexivity was framed as an ongoing practice that underpinned our transdisciplinary education work.

Reflexivity does not simply emerge by completing project tasks (Kligyte et al., 2021). Drawing on past experiences, a well-considered methodology was implemented to facilitate the reflexive process. The intent to carry out a research project alongside the practical tasks of creating PIEx in and of itself prompted us to make space for reflexivity, drawing attention to how the different types of knowledges and epistemologies were integrated in our work. Research was conducted in accordance with ethical standards following Institutional Human Research Ethics Approval, with the lead industry partners agreeing to take part in this research. Of 63 students undertaking the subject, 14% (n = 9) gave their consent for their online and workshop contributions to be included in research.

**Fig. 1:**
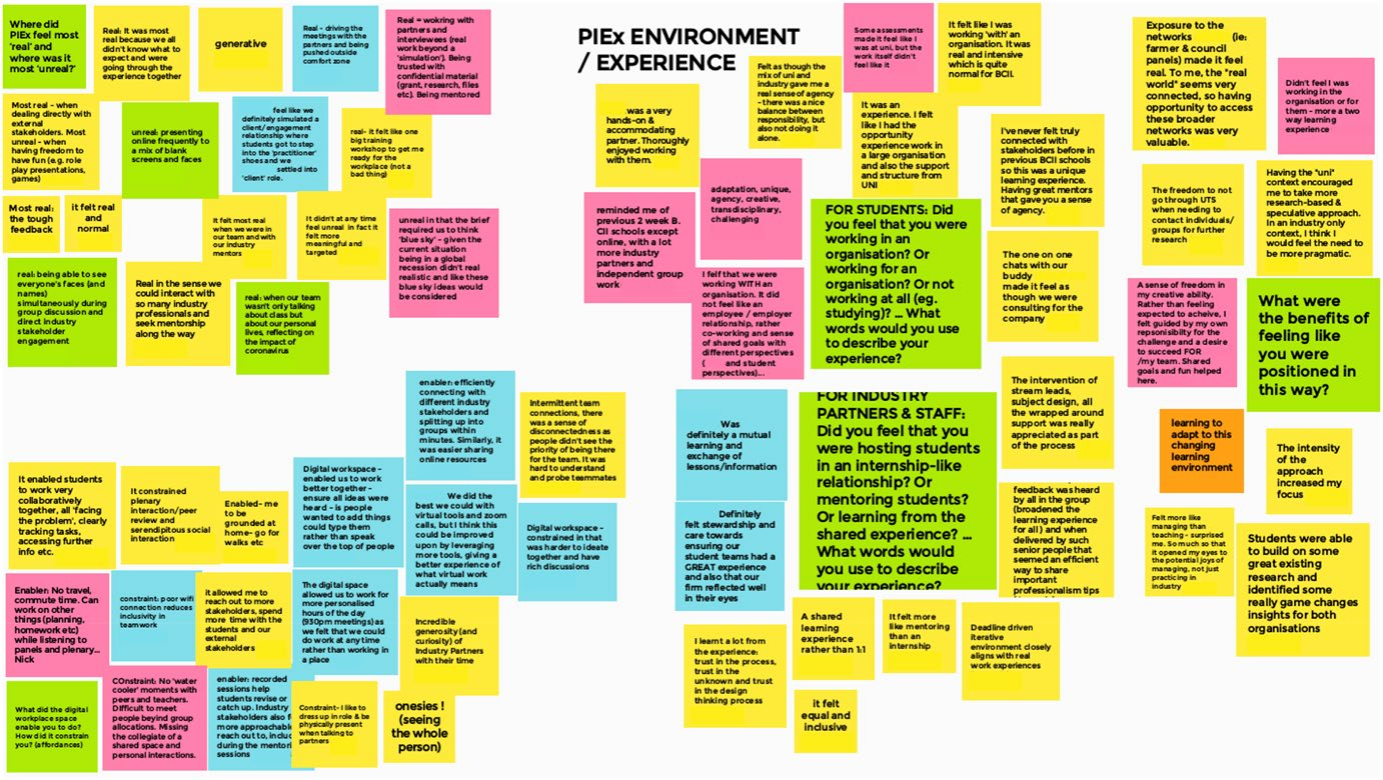
Debrief workshop participant input on an interactive online whiteboard

The key reflexive moments that generated the data examined in this paper include: (1) a debrief workshop at the conclusion of PIEx; (2) structured engagement with curated readings on liminality and education work by the team of authors; and (3) written reflections and reflexive dialogue examining PIEx through the lens of liminality and third space. The workshop involved academic staff and industry partnership team members, key industry partners and students undertaking the subject. The workshop was conducted via online conference, utilising interactive online whiteboards (see Fig. 1). The session was audio recorded and transcribed.

After the workshop, the team of authors read the transcript and engaged with curated readings on liminality, third space in education and education work (Engels-Schwarzpaul & Emadi, 2011; Land et al., 2014; Newman et al., 2014). Drawing on the readings and building on past experiences of guided reflexive processes (see Kligyte et al., 2019, 2021), we co-created a set of questions and conversation prompts. Individual written reflections were shared prior to coming together for a synchronous reflexive dialogue session, where building on the methods of ‘parlour talk’ (Werder et al., 2010) and ‘story groups’ (Labonté, 2011), we took turns to explore aspects of liminality discussed in the literature that we recognised in our practice. We also took care to notice silences and absences, with regard to characteristics of liminality, in comparison to liminal experiences in our practice. Individual perspectives were built on and extended through several iterations of a ‘reflection circle’ (Labonté, 2011). The dialogue was transcribed by one of the authors who took the role of a note-taker. Each author then edited the transcript to ensure that the key messages were accurately captured. Extracts from this dialogue are included in text boxes throughout this paper to include the authentic authorial voices and to bring the more conceptual points to life.

## Liminal WIL experiences in third space

In the debrief workshop, we sought to create an environment akin to third space, where insights and discoveries gained through PIEx could be shared by students, industry partners and academic and professional university staff members, as equals. The workshop participants were invited to explore three types of liminality: (1) liminality inherent in virtual work and learning environments during the pandemic; (2) liminality and fluidity of roles in third space, where academics, students and industry partners generate new knowledge and practices together; and (3) liminality intrinsic to transdisciplinarity which seeks to transcend disciplinarity to engage authentically with real-world challenges (Klein, 2004; McGregor, 2017). The following sections of the paper include the insights gained through this process.

### Liminality in a virtual environment

Since the PIEx offering was put together partly to mitigate the risk that a traditional internship experience undertaken remotely would feel less authentic and less engaging for students, the workshop participants were invited to consider how ‘real’ the virtual PIEx experience felt. The initial framing of PIEx was that of a simulation of a workplace, and we wanted to explore how the PIEx participants experienced this engagement: Did the students feel like they were working in an internship-like setting or studying at the university? Did the industry partners feel like they were hosting students in their workplace or mentoring them in an academic setting?

Somewhat counterintuitively, students reported that the shift to online WIL enabled them to feel more connected to the challenge and the industry partners, with some students commenting that PIEx felt “more real than most of their past BCII experiences” and “almost too good to be true”. Due to the pandemic, students, industry partners and university staff members were already grappling with the efficacy of remote study and work practices. Negotiating the unsettling circumstances of being ‘together apart’ was considered a shared liminal experience.

**Table:** 

The students were literally in a liminal space—they were at home, university and a workplace simultaneously, and so were we (academic and partnerships team), as well as the industry partners. The home had become their workplace. It wasn’t real, but yet it reflected the reality of the time—the fact that we all had to work from home. The things that we faked were almost making it more real, because of the situation we were in. The students were pretending to be graduates, but the work was real and had real outcomes. You needed the imagined bit, and you needed students to know it was imagined, in order to have the real bits working. (Beth, Work Integrated Learning Partnerships Manager)	

Being virtually present while physically distanced created an expansive learning space, enabling students to engage with external networks that would have stayed inaccessible in face-to-face settings. Several students commented that PIEx provided “a bit more than what you might get as a grad in your first job”. Whilst they missed out on “working on the floor side-by-side with other people”, students connected with and had numerous opportunities to speak to multiple stakeholders mediated by online technologies. The hard work and generosity of industry partners in opening up their networks was noted several times by university staff members. According to one industry partnership team member, through exposure to the organisational recruitment and work planning practices students were given a holistic view of the organisation. By witnessing “how organisations operate and what they care about”, students experienced a “flattening of the hierarchies”, which typically would not occur in a traditional work placement.

Students reported spending extensive time on projects in their teams, without direct supervision by industry partners or academic staff members. However, in the workshop, it became apparent that to some industry partners, the PIEx experience was primarily mediated by scheduled video conferencing sessions, with asynchronous work across the distributed platforms being relatively invisible.

**Table:** 

There were so many opportunities to make connections. Students were being connected to different areas and zones. But partners weren’t connected into all the different areas, as the students were. The people who benefitted most were those who were present throughout. […] I’m seeing a transformative liminal space for those present throughout and I’d question whether the industry partners were having a liminal experience. (Adrian, Subject Coordinator)	

These contrasting perceptions highlight the importance of collective sense-making and reflexive negotiation of what constitutes meaningful WIL by the various stakeholders. If the liminal space of PIEx is conceptualised as a space for “connection” rather than “containment” (Land et al., 2014, p. 4), the question of whether the interactions take place virtually or physically becomes less relevant. What is more important is how the design of WIL creates an environment that facilitates the unfolding of connections between different spaces, locations and people.

### Fluidity of roles in third space

The workshop participants were also invited to explore how the roles of students, industry partners and teachers were positioned in PIEx by examining who ‘owned’ the PIEx space. Did the various participants feel like they were going into somebody else's territory? If so, whose territory was it? And how did it feel to be there?

One industry partner felt that throughout PIEx they had welcomed students and university staff members into their space to share their “thought leadership and knowledge”. Conversely, university staff members felt that they invited the industry partners into PIEx as an education space. Finally, from the student perspective, they felt that staff members and industry partners joined them in their team’s space to give information and feedback about their projects. The aforementioned industry partner noted that the debrief workshop itself felt the most like the university space, offering him “the least comfort [he’s] had in the entire program”. In contrast, another industry partner reflected on the “learning from [students’] thinking and from their journey” and saw the research component as an important part of “extremely stimulating knowledge exchange”. This suggests that the liminal space of PIEx has opened up a range of perspectives on meaningful WIL by inviting each participant to negotiate their role in this “interstitial realm” (Engels-Schwarzpaul & Emadi, 2011, p. 1).

Industry partners concurred that students brought a fresh perspective to the challenges they posed, which helped them discover potential new areas of exploration. One of the stream leaders who was employed in an academic capacity in PIEx, but who otherwise works in similar settings as a consultant, also reflected that “the learning part is half the reason why [he is] doing it”. He felt that he often discovered new industry-relevant tools and ideas through students who “always surprised [him] with the ways they applied methods”.

**Table:** 

How do we shift the dial and make that space more mutually learning-focussed and primed for boundary crossing? […] It is about creating a culture where it’s okay to learn from someone half your age. There is a block when there is an expert who feels they have to impart their knowledge and already has the answers (Amanda, Strategic Partnerships Director).	

**Table:** 

Partners are co-creating our liminal space with us. We asked industry to tell us what their rituals and documents looked like, and then the stream leaders made PIEx materials based on this. Industry partners were co-creators, not just observers. We cannot control this or create a liminal space for them, but there is a third space that requires them to be involved and we cannot create these types of WIL experiences without them. (Sabrina, Industry Partnerships Officer)	

Many students enjoyed the liminality of their role in PIEx. They felt that online interactions were “less intimidating” and helped them to “speak up in front of everyone” in comparison to being in front of “a big crowd when you're in person”. Students also noted that being affiliated with a partner organisation resulted in an “increased level of respect in both directions”, which helped students broker interactions with external stakeholders:

It was sort of like, ‘Hi, I'm a student from, I'm working with [this industry partner], we are pursuing this grant and we are acting in the capacity as an innovation consultant’, rather than, ‘Oh, do you have three minutes to help me with my uni project?’ (Student participant)

By leaving the question of ‘ownership’ of PIEx open and unresolved, the participants were invited into third spaces where different participant identities, practices and temporalities were able to be negotiated. The abundance of perspectives shared at the workshop indicates that each PIEx participant was transformed in some way, although the specific learning outcomes were different for everyone.

### Liminality in transdisciplinarity

**Table:** 

The BCII magic happens when our disciplines and our people come together. All the knowledge in the room evolves. It's a profound kind of emergence that happens; you can't predict it, you can't know the outcomes in advance. That's because we’re playing with the liminality between disciplines and also between ontologies and epistemologies. So, ontology and epistemology of our learners come together in unique ways in the moment of encounter. We cannot foresee what will emerge and that's what makes teaching into this degree such a surprise and delight. (Bem, Course Director)	

Transdisciplinarity introduces another layer of complexity for those involved in creating and delivering WIL experiences within the BCII program. The BCII curriculum is built to develop students’ capacity to recognise how their own perspectives (e.g., disciplinarity, worldviews, culture and upbringing) shape their responses to the world. Students are invited to bring their whole selves to the collective learning and to carve out a unique pathway for themselves, in conversation with their peers and external stakeholders. By focusing on their passions, strengths and interests, students combine disciplinary knowledges from across fields and industries to create genuinely new responses to the most pressing challenges facing society today (Le Hunte & Kligyte, 2021).

**Table:** 

All of the curriculum culminates as it builds up to this type of liminality. In the BCII, this space is opened up. There is any number of destinations—everyone goes through their own journey to become something different. And the knowledge that emerges in this liminal space is not known already. (Giedre, Senior Lecturer in Transdisciplinary Education)	

In the debrief workshop, students and industry partners were invited to compare their PIEx experience with a traditional mono-disciplinary internship experience they might have had, either as interns or as internship supervisors. We sought examples or particular moments in PIEx where the participants noticed the value of transdisciplinary contribution.

Students appreciated the partner feedback that transdisciplinary capabilities were indeed needed in the industry, with one of the partners urging students to capitalise on the fact that they are “from these different areas of study and that has allowed [them] to develop really, really rich solutions”. However, the conversation at the workshop centred around the difficulty of communicating transdisciplinary capabilities in external contexts. There was an agreement that the notion of transdisciplinarity itself was unfamiliar and conceptually difficult to grasp. The PIEx experience stimulated students’ thinking about the need to develop appropriate ways of communicating the unique combination of their core degree with transdisciplinary expertise. Through PIEx, both students and industry partners gained new awareness about the limitations of the existing graduate recruitment systems, which filter out students’ transdisciplinary capabilities. As students noted, by ticking the ‘other’ capabilities checkbox in an online form, they “don't even have a space for [their transdisciplinary expertise] to be considered”, as they are simply unable to progress beyond the first phases of traditional recruitment. Thus, students lamented having to rely on their core degree alone to secure interview opportunities.

While one of the PIEx industry partners jokingly interrupted the discussion saying, “We will have you”, this short exchange is reflective of similar conversations with partner organisations that occur routinely at TD School. For example, after working with a group of transdisciplinary BCII students on another innovation project, one major engineering firm realised that they had been systematically diminishing the range of talent by solely prioritising students from technical backgrounds. Recognising the value of transdisciplinary capabilities, the firm transformed their graduate recruitment program to enable a more diverse cohort of students to be selected.

By inviting the participants to explore their transdisciplinary experiences, identities, relationships and practices, PIEx created a provisional and exploratory space “where things can be re-thought [and] re-authored” (Land et al., 2014, p. 5). The PIEx example demonstrates that transdisciplinary WIL spaces can enable participants to question and negotiate the existing rules, sometimes enabling them to “undo the script of self and re-script” (Land et al., 2014, p. 5), and at other times nudging whole organisations to consider change to their practices.

### Discussion and questions arising

The liminal space of PIEx opened up new questions about the nature of transdisciplinary WIL and education work. Prompted by the discussion about the ‘ownership’ of the PIEx space, the core team continued exploring the question of risks and responsibilities involved in these types of WIL experiences. In the context of the contemporary higher education discourse that regards students as ‘paying customers’ (Marginson, 2006), we pondered upon the implications of asking students to step into the liminal space where the ‘quality’ of the encounter with industry partners cannot be guaranteed by the university educators. We also queried the risks for industry partners, whereby one of the team members reflected that although our industry partners were not ‘paying’ clients, they were dedicating their time and resources, without a contractual guarantee that an appropriate outcome was going to be delivered. However, we felt that these risks were well-mitigated in our education work. Partners typically have multiple reasons to engage—access to emerging talent, as well as opportunities for staff members to mentor, trial online collaborative tools or learn more about transdisciplinary methodologies. The specific PIEx encounter also provided partners with a chance to evaluate their graduate programs, engage new stakeholders, and carve out the time to consider in depth the challenges posed to students. Similarly, the student learning outcomes were not solely dependent on the industry partner input; they were also facilitated by teaching staff through curated learning opportunities and assessment tasks.

Through ongoing reflexive dialogue, we extended our own understanding of education work and relationships with industry partners beyond the PIEx encounter itself. One of the Industry Partnership Team members reflected on “a huge amount of work done by the partnerships team to generate relationships with our partners that are not transactional, one-off or temporary”. Indeed, relationships and trust are built over time, with the Industry Partnerships Team developing comprehensive knowledge about the different partners’ short and long-term goals. Through this, the TD School WIL offering is built upon trust, and it is centred around opportunities where both partners and students can explore interesting, complex problem spaces together. Learning in PIEx encompassed more than what was intentionally designed to be educational. The real-world problems that students were grappling with extended into partner organisations, with students’ creative responses calling into question some of the practices ingrained in partner organisations, as well as questioning the assumptions implicit in their challenges. During the pandemic, industry partners had limited capacity to question and re-think the emerging work practices outside of educational encounters like PIEx. The connection to students and their thinking energised partner organisations and, from the university staff members’ perspective, opened the door to further engagement with industry partners. We pondered upon the possibility for these types of liminal WIL experiences to become a vital link and a key contribution to external communities by universities serving their public mission.

Similarly, the liminal space of PIEx opened up opportunities for the university staff members to reconsider their own practices. The exploration of the authenticity of the PIEx WIL experience led the subject coordinator to reflect that although PIEx was not a ‘real’ professional experience in a traditional sense, it was a “real learning experience: we were stepping into the unknown with real partners, people and problems and it… felt meaningful”. The subject coordinator highlighted the importance of working with existing student expectations from the outset: “what are they expecting? Are they expecting an internship? A professional role in an organisation? Or are they expecting a learning experience which connects them to the professional world?” Opportunities to explicitly consider and reframe the preconceptions about the purpose of a WIL experience are often missing in traditional WIL settings. By encouraging the participants to pose and consider integrative ideas in conversation with the various stakeholders, PIEx enabled the participants—students, industry partners and university staff members—to collectively envisage, reimagine and adapt their practices.

An important consideration raised by one Industry Partnerships Team member was the question of inequality in these types of liminal WIL experiences. Discussing the risk of inadvertently excluding those who do not possess the right language or creative methodologies, this team member reflected that “liminal spaces are easier to navigate for students who are more supported—or privileged”. Indeed, she herself “didn’t instantly get [transdisciplinarity] and is constantly being provoked as there are so many methodologies that aren’t part of [her] education”. Being engaged in transdisciplinary education work beyond this project, there is a shared desire by the PIEx team to be continually challenged to reconsider our practice. The reflexive process alongside the practical tasks of creating the PIEx experience, in particular, has helped us to create a liminal learning space where our thinking and practice can “stay emergent and fresh” (Land et al., 2014, p. 1).

## Concluding remarks

Building on the tradition of transdisciplinarity, staff members at TD School routinely involve students in the co-creation of their educational experiences (Baumber et al., 2020). Whilst this paper contributes to the literature on curriculum co-creation (for example, see Mercer-Mapstone et al. (2017), it expands this scholarship beyond student-facing learning experiences alone. Instead, transdisciplinary education work is conceptualised as taking place in between disciplines, roles and organisations with multiple benefits for diverse participants. Through engagement with the conceptual lenses of liminality and third space, this study contributes a further novel perspective to the literature on education work. We demonstrate how by inviting the participants into third space—the WIL encounter in which roles, identities and rules of engagement are intentionally uncertain—education work becomes less about delivering ‘quality’ WIL and more about creating “realm[s] of pure possibility whence novel configurations of ideas and relations may arise” (Turner, 1967, p. 97). Specifically, we identify the reflexive engagement in collective intention-setting and sense-making as a key enabler of liminal WIL experiences like PIEx, calling participants to challenge, reframe and mould their reasons for engagement in and understanding of WIL. Through this, the purpose of WIL engagement itself can be reframed from being primarily about student learning to that of an evolving and inclusive educational encounter in third space where all participants can gain mutual value.

Finally, through a reflexive process outlined in this paper, we, the university staff members, have become more aware about our own education work and how we ourselves can be transformed through participation in liminal third space. For example, in PIEx, we had an opportunity to witness the expected and unexpected learning outcomes for both students and industry partners in a way that would not have been possible in traditional internship settings. We have also become more cognisant about the expertise and complex processes involved in our own practice. The work of engaging with a range of variously positioned players in order to notice, broker, negotiate, assemble, translate, re-imagine, and re-invent opportunities, as well as communicate the value and impact of these types of educational initiatives to a range of stakeholders, is neither straightforward nor simple. The reflexive process carried out as part of PIEx enabled us to articulate aspects of our work in new ways, inviting us to notice the imaginative and creative aspects of this relational work. These insights are likely to continue having impact on TD School staff, students and industry partners, long after the pandemic, leading to new opportunities and further avenues for action.

We encourage other scholars and practitioners engaged in education work to probe and articulate their practice so that the complexities involved in working across the porous boundaries of contemporary universities can be understood better, for the possibilities that are opened up, as well as the risks that are entailed in this type of emergence. Whilst we found the concepts of liminality and third spaces fruitful, there are many other ways to enact and inquire into liminal learning experiences. In particular, with the pedagogical responses to the pandemic becoming more normalised, there is a need to consider how we can learn from the rapid pandemic pivot to online teaching, so that exciting and engaging liminal learning experiences can be designed in more stable times to include more fluid, emergent, creative and transformative opportunities for learning beyond the ‘contained’ traditional parameters of educational delivery. Finally, with an increasing need for universities to demonstrate social impact as a core aspect of their endeavour, further research is needed to understand more fully how these types of liminal experiences can contribute to forging and sustaining productive relationships in broader innovation ecosystems, leading to real impact in the world and a refreshed vision for the purpose of a university.
